# Inflammation interferes with chemoreception in pigs by altering the neuronal layout of the vomeronasal sensory epithelium

**DOI:** 10.3389/fvets.2022.936838

**Published:** 2022-09-12

**Authors:** Violaine Mechin, Pietro Asproni, Cécile Bienboire-Frosini, Alessandro Cozzi, Camille Chabaud, Sana Arroub, Eva Mainau, Patricia Nagnan-Le Meillour, Patrick Pageat

**Affiliations:** ^1^Tissue Biology and Chemical Communication Department, IRSEA, Institute of Research in Semiochemistry and Applied Ethology, Apt, France; ^2^Molecular Biology and Chemical Communication Department, IRSEA, Institute of Research in Semiochemistry and Applied Ethology, Apt, France; ^3^Research and Education Board, IRSEA, Institute of Research in Semiochemistry and Applied Ethology, Apt, France; ^4^Statistics and Data Management Service, IRSEA, Institute of Research in Semiochemistry and Applied Ethology, Apt, France; ^5^Department of Animal and Food Science, School of Veterinary Science, Universitat Autònoma de Barcelona, Barcelona, Spain; ^6^Univ. Lille, CNRS, INRAE, UGSF - Unité de Glycobiologie Structurale et Fonctionnelle, Lille, France

**Keywords:** vomeronasal organ, inflammation, chemodetection, chemical communication, pig

## Abstract

Chemical communication is widely used by animals to exchange information in their environment, through the emission and detection of semiochemicals to maintain social organization and hierarchical rules in groups. The vomeronasal organ (VNO) is one of the main detectors of these messages, and its inflammation has been linked to behavioral changes because it potentially prevents molecule detection and, consequently, the translation of the signal into action. Our previous study highlighted the link between the intensity of vomeronasal sensory epithelium (VNSE) inflammation, probably induced by farm contaminant exposure, and intraspecific aggression in pigs. The aim of this study was to evaluate the cellular and molecular changes that occur during vomeronasalitis in 76 vomeronasal sensorial epithelia from 38 intensive-farmed pigs. Histology was used to evaluate the condition of each VNO and classify inflammation as healthy, weak, moderate, or strong. These data were compared to the thickness of the sensorial epithelium and the number of type 1 vomeronasal receptor cells using anti-Gαi2 protein immunohistochemistry (IHC) and analysis. The presence of odorant-binding proteins (OBPs) in the areas surrounding the VNO was also analyzed by IHC and compared to inflammation intensity since its role as a molecule transporter to sensory neurons has been well-established. Of the 76 samples, 13 (17%) were healthy, 31 (41%) presented with weak inflammation, and 32 (42%) presented with moderate inflammation. No severe inflammation was observed. Epithelial thickness and the number of Gαi2+ cells were inversely correlated with inflammation intensity (Kruskal–Wallis and ANOVA tests, *p* < 0.0001), while OBP expression in areas around the VNO was increased in inflamed VNO (Kruskal–Wallis test, *p* = 0.0094), regardless of intensity. This study showed that inflammation was associated with a reduction in the thickness of the sensory epithelium and Gαi2+ cell number, suggesting that this condition can induce different degrees of neuronal loss. This finding could explain how vomeronasalitis may prevent the correct functioning of chemical communication, leading to social conflict with a potential negative impact on welfare, which is one of the most important challenges in pig farming.

## Introduction

Chemical communication plays a key role in animal life as it ensures the exchange of semiochemical information between subjects of the same or different species. This type of intraspecific or interspecific communication is performed through the exchange of chemical signals in all aspects of animal life, including maternal recognition, reproduction, territorial marking, and predatory/prey recognition (1–3). These chemosignals are composed of molecules of different nature that are released by the emitting animal (through biological fluids or secretions, scent marks, etc.) and are detected by sensory organs, such as the accessory olfactory system, and especially the vomeronasal organ (VNO) (3–5). Also, the main olfactory system is known to attribute excellent olfactory abilities to most mammals as in reproduction or in social responses (6–8) due to to a large and organized olfactory structure (9, 10). Concerning the VNO, this tubular and bilateral organ is located in the nasal cavity of most animals and is composed of a non-sensory epithelium (NSE) and a sensory epithelium (vomeronasal sensory epithelium (VNSE)) arranged around a lumen where the environmental air containing semiochemicals transits (11, 12). In most mammals, these molecules are detected by vomeronasal sensory neurons expressing type 1 (V1R) or type 2 (V2R) receptors, distinguishable by their respective coupling with Gαi2 or Gαo proteins in the cytoplasm for further signal transduction (13) and by formyl peptide receptors (FPRs) supposed of acting as chemosensory receptors in the mouse VNO (14).

The key role of this organ in animal life has been ascertained by investigating behavioral modifications after induced VNO changes, which can provoke alterations in social, maternal, or sexual behaviors in different species (15–19). Asproni and colleagues revealed that the presence of VNO spontaneous inflammation (vomeronasalitis) was also associated with intraspecific aggression in cats (20). This link was recently confirmed in a stable social group of pigs, in which a strong correlation was found between the histological scores of VNO inflammation intensity and the number of skin lesions induced by social fighting (21).

In fact, the animal social structure is composed of a set of behaviors, as in pigs or wild boars, in which a basal level of aggression always exists, even in stable social groups, to ensure hierarchy rules (22). In pigs, signs of aggression are linked to long-term social stress, which impacts their welfare and productivity (23, 24). To date, the link between vomeronasalitis and behavior has been verified (21).

The purpose of the present study was to investigate the cellular changes that occur in the sensory epithelium of the VNO when inflamed to unveil how vomeronasalitis can alter chemical communication capabilities, impacting the behavior of the affected animals, which could also induce a decrease in their welfare.

The repartition of the three types of receptors (V1Rs, V2Rs, and FPRs) has been proven to be highly modified between species (25, 26). In pigs, genomic analyses detected only the presence of functional V1Rs, in contrast to V2Rs and FPRs (27). Similar to that in other species, these results indicate that the V1R gene family is responsible for semiochemical detection in pigs (27).

Histology was used to assess the condition of each VNSE (healthy, weak, or moderate inflammation), which was compared to its thickness since these changes have already been shown in olfactory mucosa inflammation (28, 29). Gαi2 protein was studied by immunohistochemistry (IHC) to identify V1R expressing cells, the main receptor type in pigs. Finally, the expression of the porcine odorant-binding protein (OBP) was evaluated by IHC, since these small soluble proteins secreted in the mucus are required to permit signal transmission in the receptor cells (30, 31).

## Materials and methods

The VNO samples used in this study were collected from a large project approved by the Institutional Animal Care and Use Committee of the Institute of Agrifood Research and Technology (IRTA) and Generalitat de Catalunya (protocol number 7622).

### Animals and sampling procedures

This study included 76 VNOs sampled from 38 6-months-old female pigs ([Landrace × Large White] × Piétrain), corresponding to a population used for porcine VNO in a previous study (21) and to the common age of slaughtering, which allows having a representative image of what happens in a farm pig VNO during its productive cycle. They were maintained in the Institute of Agrifood Research and Technology (IRTA, Monells, Spain) facilities in slatted pens (5 × 2.7 m) with water and food *ad libitum*. At 23 to 27 weeks of age (mean 108.0 ± SD 12.4 kg of body weight), they were exposed to a 90% CO_2_ stunner for 3 min before exsanguination. Immediately after death, snouts were collected and immersed in 10% buffered formalin (pH 7.4) until complete tissue fixation.

After their extraction from the nasal cavity, the 76 VNOs were trimmed into 2–3 mm thin sections and dehydrated and paraffin-embedded according to routine histological methods. Sections (3.5 μm thick) were cut and dried overnight at 37°C on SuperFrostPlus™ slides (Cat No. 10149870, Thermo Fisher Scientific, Illkirch, France) before being subjected to histological and immunohistochemical analyses.

### Histopathological analysis

Each VNO was stained with hematoxylin and eosin (H&E, BioOptica, Milan, Italy) to classify VNSE inflammation intensity on a scale from 0 to 3 (0 = absence of any sign of inflammation: healthy epithelium, 1 = weak inflammation; 2 = moderate inflammation, 3 = strong inflammation), as previously reported (21).

**Figure 1:**
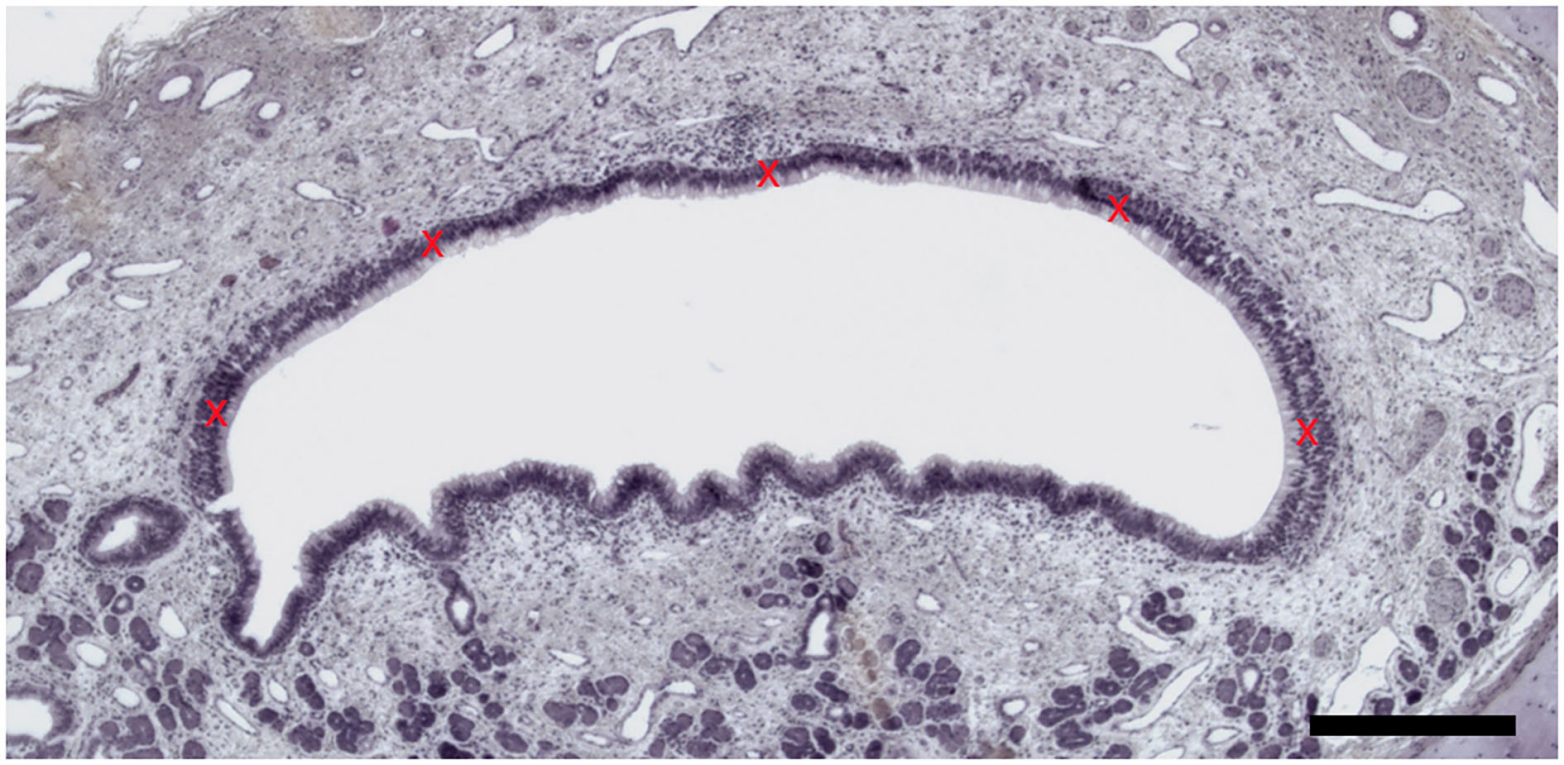
Vomeronasal sensory epithelium thickness measurement. Five measures (red crosses) were taken by VNSE. (Hematoxylin and eosin (H&E) staining, Objective x10, Scale bar 100 μm).

Hematoxylin and eosin (H&E)-stained sections were also used to measure the VNSE thickness. Microscopical pictures were taken with the microscope EVOS® FL Auto Imaging System (Thermo Fisher Scientific, Illkirch, France) and its software and measurements were obtained with the software Image J (US National Institute of Health, MD, USA) (32) on five different parts of the VNSE, starting from the basement membrane to the top of the knobs, as described in previous studies focused on mouse olfactory mucosa inflammation (33). These measurements were repeated on three different representative sections of each VNO to obtain a mean value (*N* = 15), which is considered the definitive VNSE thickness, expressed in micrometers. The VNSE thickness measurement of the five parts follow the scheme illustrated in Figure 1.

### Immunohistochemical analysis

After deparaffinization and rehydration, VNO sections were subjected to microwave antigen retrieval in 0.1 M citrate buffer pH 6 solution (Cat No. F/T0050; DiaPath SpA, Martinengo, Italy) at 560 W for 3 min and 30 s, followed by 15 min at 210 W. Endogenous peroxidase blocking was performed using 100 μl of peroxidase blocking solution (Cat No. ACA500; Scytek, Logan, UT, USA) for 30 min. The sections were rinsed and incubated for 1 h at room temperature with the primary antibodies. Anti-Gαi2 antibody (rabbit polyclonal, Cat No. sc-7276; Santa Cruz Biotechnologies, Dallas, TX, USA) was diluted at 1:200, and anti-porcine OBP [rabbit polyclonal, provided by Dr P. Nagnan-Le Meillour's laboratory (30)] was diluted at a ration of 1:10,000. IHC anti-Gαi2 protein was performed to identify and count V1Rs expressing neurons in the VNSE, and IHC was performed to identify the presence of OBP in the area surrounding the VNO. The slides were rinsed in Tris-Buffered-Saline (TBS)–Tween 1:100 and incubated with a secondary biotinylated anti-rabbit antibody (Cat No. T/ABE125; UltraTek, ScyTek Laboratories, Logan, UT, USA) for 10 min at room temperature. Finally, streptavidin-peroxidase (Cat No. 12694067, Invitrogen, Carlsbad, CA, USA) was applied to the slides for 10 min, and visualization was performed using 3,3'-diaminobenzidine tetrahydrochloride (ImmPACT® DAB Peroxidase Substrate, Cat No. SK4105; Vector Laboratories, Burlingame, CA, USA) and counterstained with hematoxylin for 2 min. The tissues were dehydrated, cleared in xylene, and mounted. As a negative control, the primary antibodies were replaced with non-immune rabbit serum.

Slides were observed using the EVOS® FL Auto Imaging System (Thermo Fisher Scientific, Illkirch, France) and images were obtained for further analysis. Concerning the Gαi2 protein investigation, IHC-positive cells were counted with ImageJ® software on the total surface of the VNSE and were then converted to a number of positive cells per 1 mm^2^ of VNSE.

Odorant-binding protein (OBP) positivity was obtained using ImageJ® software and its color deconvolution plugin to measure stained pixels corresponding to the presence of OBP in the total VNO section. We obtained a measure expressed as the percentage of positivity in the entire VNO soft tissue of each sample.

### Statistical analysis

Data were analyzed using the Statistical Analysis System (SAS 9.4 software 2002–2012; SAS Institute Inc., Cary, NC, USA). The significance threshold was set at 5%.

Each VNO was analyzed individually and classified according to the VNSE inflammation score. VNSE thickness, Gαi2, and OBP protein expression were independently analyzed according to the inflammation score. First, the normality of each parameter was verified using the UNIVARIATE procedure, and second, homoscedasticity was checked using the Global Linear Model (GLM) procedure.

Concerning the VNSE thickness, normality and homoscedasticity were not verified; therefore, a non-parametric alternative Kruskal–Wallis test was used with the NPAR1WAY procedure. Multiple comparisons were obtained by computing the Wilcoxon tests for each pair of modalities of VNSE alteration scores. Bonferroni correction was applied using the MULTTEST procedure to control for type I errors.

For the Gαi2 protein, normality and homoscedasticity were verified, and conditions were satisfied by applying a one-way ANOVA with the GLM procedure. Multiple comparisons were performed using Tukey–Kramer adjustment by adding the LS MEANS statement to the procedure. The correlation between the Gαi2 protein levels and VNSE thickness was explored using the CORR procedure. As normality was not verified for these parameters, a Spearman coefficient (Rhô) was used to measure the possible correlation that may exist between them. With regard to the OBP parameter, normality and homoscedasticity were not verified. Consequently, the non-parametric alternative Kruskal–Wallis test was used, followed by the Wilcoxon two-sample test with a Bonferroni correction for multiple comparisons.

## Results

Of the 76 VNO samples from 38 intensive-farmed pigs, 13 (17%) were healthy, 31 (41%) presented with weak inflammation, and 32 (42%) had moderate inflammation. No severe inflammation was observed. An inflammatory infiltrate composed of small lymphocytes was observed in the soft tissue above the sensory epithelium. Small quantities of plasma cells and macrophages, such as rare mast cells and non-degenerate neutrophils, were present. Neutrophils were exclusively located in the epithelium. Moderate inflammation presented as inflammatory cells infiltrating the vomeronasal nerves and glands. The descriptive data for all the parameters are shown in Table 1.

**Table 1:** Mean, standard deviation (STD DEV), standard error (SE), and median (MED) of epithelium thickness, Gαi2+ cells, porcine odorant binding protein (OBP) according to the vomeronasal organ (VNO) inflammation score as 0 = absence; 1 = weak; 2 = moderate; 3 = strong inflammation.

	**VNO inflammation**	***N***	**MEAN**	**STD DEV**	**SE**	**MED**
Epithelium thickness (μm)	0	13	93.30	5.91	1.64	91.85
	1	31	74.16	12.87	2.31	73.01
	2	32	65.22	11.14	1.97	61.32
Gαi2+ cells (nb/mm^2^)	0	13	802.38	98.01	27.18	796.00
	1	31	485.65	107.95	19.39	477.00
	2	32	358.00	145.45	27.71	347.50
OBP (% positivity)	0	13	0.95	0.37	0.10	0.87
	1	31	7.02	7.07	1.27	5.39
	2	32	6.44	6.36	1.12	3.94

### Thickness of the epithelium

**Figure 2:**
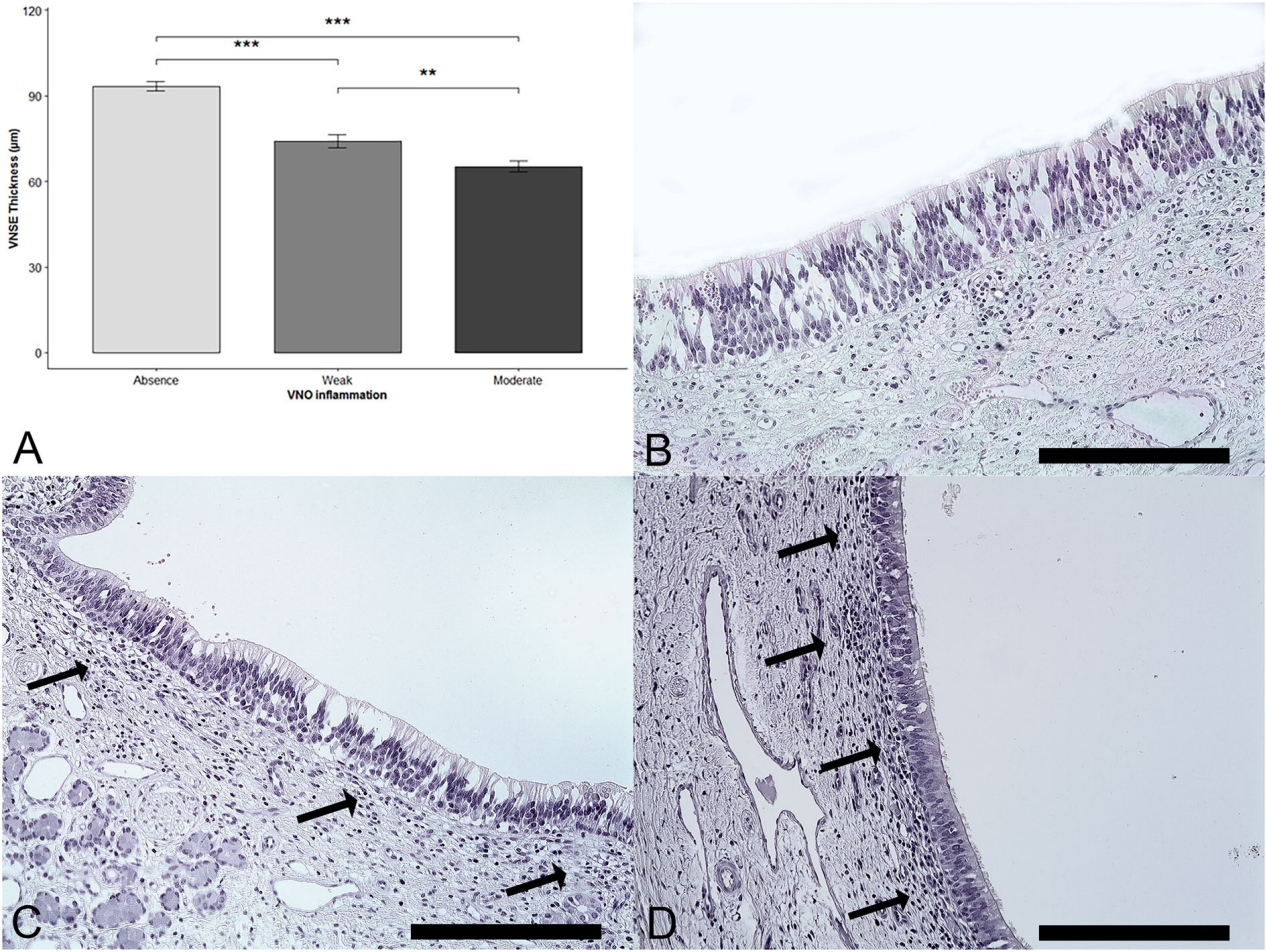
Vomeronasal sensory epithelium (VNSE) thickness decreases with inflammation intensity. Hematoxylin and eosin (H&E) staining was used to measure the thickness. **(A)** VNSE thickness according to the vomeronasal organ inflammation. Data are expressed in μm and shown as the mean ± SD (*** = *p* < 0.001; **: *p* < 0.01). **(B)** Healthy epithelium, score = 0, **(C)** Weak inflammation, score =1: few lymphocytes were found under the epithelium (black arrows). **(D)** Moderate inflammation, score =2: denser inflammatory infiltrate mainly composed of lymphocytes was found above the VNSE (black arrows). (Objective x20, Scale bars 200 μm).

The thickness of the sensory epithelium was compared with the degree of inflammation. A significant effect of the VNSE inflammation score was observed (DF = 2; χ^2^ = 31.39; *p* < 0.0001; Kruskal–Wallis test). The multiple comparisons after the Bonferroni correction showed a significant decrease in thickness when the VNSE inflammation intensity was increased, between those that were healthy and those that were weakly inflamed (*p* = 0.0003); between those that were healthy and those that were moderately inflamed (*p* = 0.0003); and between those that were weakly and those that were moderately inflamed (*p* = 0.0033) (Figure 2).

### Quantity of Gαi2+ cells

**Figure 3:**
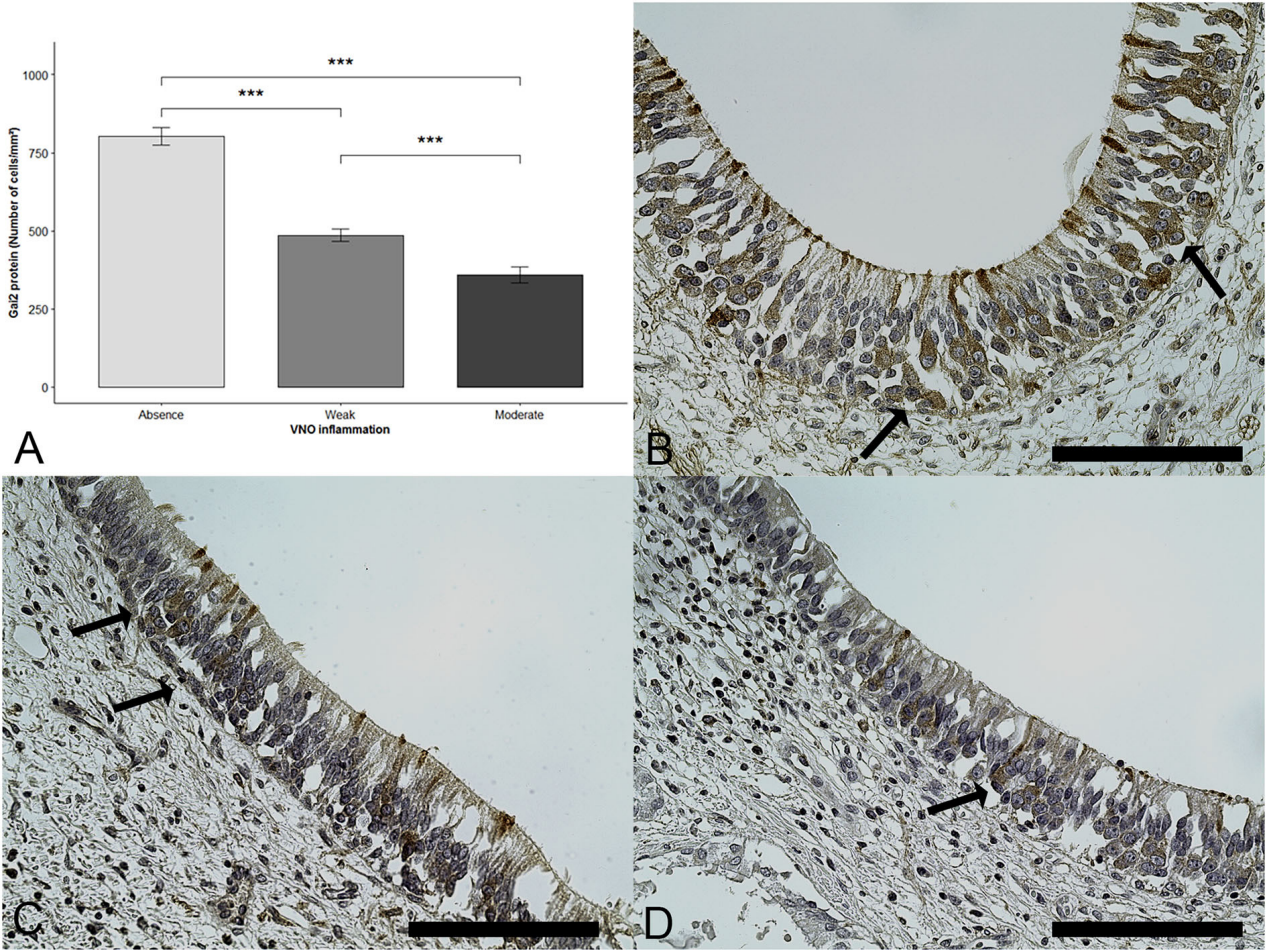
Gαi2+ cells number decrease with inflammation intensity. Immunohistochemical staining was used to reveal the presence of Gαi2 protein (brown staining shown by arrows). **(A)** Mean and standard error of the Gαi2 protein according to the vomeronasal organ inflammation. Data are expressed in number of Gαi2+ cells /mm^2^ and shown as the mean ± SE (*** = *p* < 0.001). **(B)** Healthy epithelium, score = 0, **(C)** Weak inflammation, score =1, **(D)** Moderate inflammation, score =2. (Objective x40, Scale bars 100 μm).

Statistical analyses showed that, when the epithelium was inflamed, the number of Gαi2+ cells was significantly reduced (DF = 2; Fisher Test (F) = 59.46; *p* < 0.0001; one-way ANOVA). Multiple comparisons revealed a significant difference between the VNSE scores of 0 and 1, between the VNSE scores of 0 and 2 (*p* < 0.0001 each), and between the scores of 1 and 2 (*p* = 0.0003), as presented in Figure 3.

A moderate positive correlation between the thickness and quantity of Gαi2 protein expression was observed (rho = 0.62, *p* < 0.0001; Spearman's test).

### Odorant-binding protein

**Figure 4:**
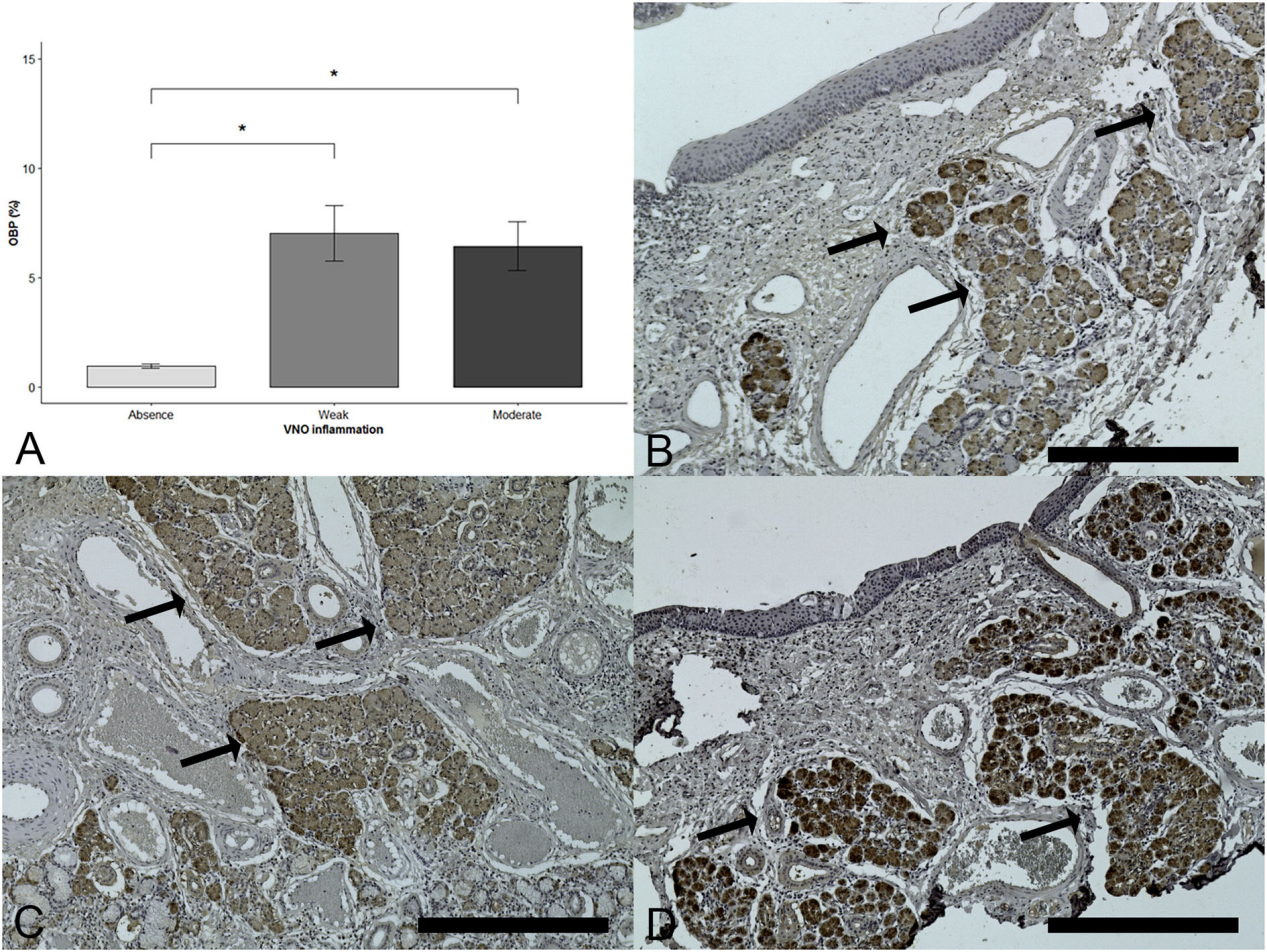
Odorant-binding protein (OBP) increases with the presence of inflammation in the vomeronasal sensory epithelium. Immunohistochemical staining was used to reveal the presence of OBP (brown staining shown by arrows). **(A)** Mean of the OBP protein according to the VNO inflammation. Data are expressed in percentages of OBP expression positivity and shown as the mean ± SD (* = *p* < 0.05). **(B)** Healthy epithelium, score = 0, **(C)** Weak inflammation, score =1, **(D)** Moderate inflammation, score = 2. (Objective x10, Scale bar 400 μm).

Concerning the presence of OBP in the area surrounding VNSE, protein expression was significantly increased when VNSE was inflamed (DF = 2; χ^2^ = 9.34; *p* = 0.0094; Kruskal–Wallis test). Multiple comparisons (with Wilcoxon two-sample tests) indicated that a difference was obtained between grade 0 and the presence of inflammation in grades 1 and 2 (*p* = 0.0135 each), with no difference between grade 1 and grade 2 (*p* = 0.9616) (Figure 4).

## Discussion

Epithelium inflammation has been studied in the human and mouse olfactory epithelium and has been shown to induce a reduction in epithelium thickness and alterations in olfactory capabilities, such as hyposmia and anosmia (28, 29, 34). It occurs because of many factors, including environmental contaminants such as organic dust or natural pollutant gases such as ammonia or hydrogen sulfide (35, 36). Since the sensory epithelium of the VNO is similar to the olfactory epithelium from a morphological and functional point of view (2), we can suppose that the pollutants that induce rhinitis and olfactory mucosa inflammation induce inflammation of the VNO. Furthermore, it is well-known that the concentration of these environmental contaminants increases under intensive farm conditions because of the high animal density (37, 38). However, the impact of environmental contaminants on the occurrence of vomeronasalitis requires further investigation.

Vomeronasalitis has been described in cats and recently in pigs, and in both cases, it was associated with intraspecific aggressive behavior (20, 21). However, there is a lack of data on the cellular and molecular characterization of this pathology. Our study provides more information on this topic, indicating that vomeronasalitis strongly impacts the VNO epithelium structure, inducing important cellular changes.

Due to the limited information available concerning the analysis of VNO inflammation and because this organ looks like the olfactory mucosa from a histological point of view (2, 3), we compared our results with the literature on olfactory epithelium inflammation (28, 34), which has been more widely studied and found to be in agreement. In fact, our results showed that inflammation of the VNSE induces a reduction in its thickness according to inflammation intensity, confirming what was reported by other studies that showed an association between the reduction of the olfactory epithelium thickness and its inflammation (29, 39). The inflammatory microenvironment composition has been shown to induce apoptosis (40–42) and olfactory mucosa epithelial thickness reduction (34, 43) and could be an aspect that will deserve to be explored to better understand our results.

To verify the impact of vomeronasalitis on neuronal layout, V1R neurons were analyzed immunohistologically. In the VNSE, it is known that the Gαi2 protein plays a major role in communication because it interacts with the V1R in charge of the detection of small organic/volatile molecules. These signals are used in social communication, as in maternal or sexual exchanges, and provide signal transduction, leading to neuronal responses following V1R activation (44–47). Their crucial role is even more important to be explored in this species, since this kind of receptor is, to date, the only one found in pig VNSE. In fact, V2Rs, typically characterized in other species such as rodents and marsupials, coupled with the Gαo protein, have never been observed in pigs and in most ungulates and carnivores (26, 27, 48–51).

Diminution or inhibition of Gαi2 gene expression has been shown to induce behavioral complications in Gαi2 mutant mice, such as modified sexual behaviors or an increase in maternal aggression (47, 52). In this study, we found that, when inflamed, VNSE possesses fewer cells expressing the Gαi2 protein, clearly suggesting a decrease in the number of V1R neurons in VNSE.

In addition to functional organization, the presence of OBPs in the VNO has been proven essential to ensuring semiochemical detection (30, 53–55). These proteins are secreted by the olfactory epithelium glands in high quantities in the nasal mucus (56, 57) and are hypothesized to be odorant transporters that deliver these olfactory molecules to receptors in pigs (58, 59), cows (53), and other mammals (60, 61). The present study showed that, when inflammation was detected in VNSE, regardless of the intensity, the expression of OBP in the surrounding areas was increased. This observation could be explained by the modulatory role of OBP in the inflammatory response proposed by Mitchell et al. (62), as this protein seems to inhibit neutrophil recruitment by inflammatory mediators in the respiratory system (62). Some studies on other species demonstrated OBP compensatory properties on olfactory systems, where different OBP subtypes have been reported to occur simultaneously (63–65). These kinds of studies should be exploited in the pig species to further analyze the effect of the sensory epithelium damages on the OBP expression and thus on the detection capabilities.

This study allowed us to better characterize vomeronasalitis in pigs and the modifications the condition induces in the VNO. Asproni et al. (21) demonstrated that, when inflammation was present in a pig's VNSE, the animal was more susceptible to aggression by congeners. Our results showed that inflamed VNSE possesses a lower number of neurons responsible for chemoreception. As already shown in the olfactory epithelium, a decreasing number of neurons induces a loss of efficiency such as hyposmia or anosmia (28, 34), driving the authors to suppose that the VNO could also be functionally impacted by this loss of neurons, inducing troubles in pig chemical communication, which is crucial in this species due to its social organization.

Under intensive conditions, the environmental air composition is harmful to the respiratory tract (37, 38) and, thus, potentially to their VNOs. Intraspecific chemical communication is fundamental in animal life, particularly in farm animals, since these animals need to use their communicative skills to better deal with restricted areas and to exchange with other animals. This study highlights the importance of the effects of vomeronasalitis in farm animals, and it can open novel perspectives focused on the limitation of its onset to improve welfare, which is strongly linked to animal behavior and communication.

## Conclusion

Our results permit the investigation of the molecular and cellular mechanisms by which inflammation of the VNO alters chemodetection in pigs, potentially contributing to the onset of aggressive behaviors in the farm pen. In fact, neuronal loss caused by the inflammatory process seems to critically reduce the chemoreceptive capabilities of the affected animals by diminishing the VNSE thickness and decreasing the number of V1R neurons. To the best of our knowledge, this study is the first to characterize the effects of vomeronasalitis on VNO function, paving the way for further in-depth studies on the link between chemoreception, animal pathology, behavior, and welfare.

This study provides new insights into the characterization of VNO inflammation and the mechanisms by which it interferes with chemoreception and animal behavior.

## Data availability statement

The raw data supporting the conclusions of this article will be made available by the authors, without undue reservation.

## Ethics statement

The animal study was reviewed and approved by Institutional Animal Care and Use Committee of IRTA and Generalitat de Catalunya.

## Author contributions

Conceptualization: PA, CB-F, PP, and AC. Methodology: VM, PA, CC, EM, and PN-LM. Validation, writing—original draft preparation, and visualization: VM and PA. Formal analysis and data curation: SA. Investigation: VM, PA, CC, and EM. Resources and funding acquisition: PP. Writing—review and editing: PA, CB-F, AC, PN-LM, PP, and SA. Supervision and project administration: PA. All authors have read and agreed to the published version of the manuscript.

## Conflict of interest

The authors declare that the research was conducted in the absence of any commercial or financial relationships that could be construed as a potential conflict of interest.

## Publisher's note

All claims expressed in this article are solely those of the authors and do not necessarily represent those of their affiliated organizations, or those of the publisher, the editors and the reviewers. Any product that may be evaluated in this article, or claim that may be made by its manufacturer, is not guaranteed or endorsed by the publisher.

## Abbreviations

FPR: Formyl Peptide Receptor
Gαi2: guanine nucleotide-binding protein G (i) subunit alpha-2
Gαo: guanine nucleotide-binding protein G (o) subunit
H&E: Hematoxylin and Eosin
IHC: Immunohistochemistry
OBP: Odorant-Binding Protein
NSE: Non-Sensory Epithelium
VNO: Vomeronasal Organ
VNSE: Vomeronasal Sensory Epithelium
V1R: Vomeronasal Receptor Type 1
V2R: Vomeronasal Receptor Type 2.
